# Isolated Cervical Lymphadenopathy in Pediatric Rosai-Dorfman Disease: A Case Report and Literature Review

**DOI:** 10.7759/cureus.100914

**Published:** 2026-01-06

**Authors:** Naima Baddouh, Khalila Nainia

**Affiliations:** 1 Department of Pediatrics, Faculty of Medicine and Pharmacy, Mohammed VI University Hospital Center, Agadir, MAR

**Keywords:** child, immunohistochemistry, lymphadenopathy, rosai-dorfman disease, sinus histiocytosis

## Abstract

Rosai-Dorfman disease (RDD) is a rare, benign histiocytic disorder that typically presents with bilateral cervical lymphadenopathy. Pediatric cases with isolated, unilateral involvement are uncommon and may pose diagnostic challenges. We report the case of an 11-year-old boy presenting with isolated, unilateral cervical lymphadenopathy without systemic symptoms. Biological and radiological investigations were nonspecific. Histopathological examination of the excised lymph node revealed characteristic large histiocytes with emperipolesis and an immunohistochemical profile consistent with RDD (CD163+, CD1a-, and CD20-). The patient was successfully managed with surgical excision alone, with no recurrence or need for additional therapy. This case highlights that RDD may present as isolated, unilateral cervical lymphadenopathy in children and can be effectively managed with surgery alone in selected cases. Awareness of this atypical presentation is essential to ensure accurate diagnosis and avoid unnecessary medical treatment.

## Introduction

Rosai-Dorfman disease (RDD), also known as sinus histiocytosis with massive lymphadenopathy, is a rare histiocytic disorder first described by Rosai and Dorfman in 1969 [1]. It was classified by the Histiocyte Society in 1987 as a non-Langerhans cell histiocytosis (LCH) [2] and is currently categorized by the World Health Organization among histiocytic and dendritic cell neoplasms [3], with an estimated prevalence of approximately 1 in 200,000 [4]. Although traditionally considered a benign and reactive condition, recent molecular studies have identified activating mutations in the MAPK signaling pathway in a subset of patients, suggesting a clonal proliferative process and reshaping the current understanding of the disease [5].

RDD most commonly affects young adults, with a slight male predominance and a higher incidence reported in individuals of African descent, whereas pediatric cases remain relatively uncommon [6]. Clinically, the disease often presents with painless cervical lymphadenopathy but may also involve extranodal sites, resulting in a wide spectrum of manifestations. In children, atypical presentations and nonspecific clinical, biological, and radiological findings may complicate the diagnostic process and raise concern for malignant conditions such as lymphoma [7].

Histopathological examination remains the cornerstone of diagnosis, characterized by large histiocytes exhibiting emperipolesis and a distinctive immunohistochemical profile [8]. Although infectious causes, including viral etiologies, have been proposed in the past, no conclusive evidence supports these hypotheses [7]. Management strategies vary depending on disease extent and symptomatology, ranging from observation to surgical or systemic therapies.

We report a pediatric case of RDD with an unusual clinical presentation, highlighting the diagnostic challenges, the importance of histopathological confirmation, and the potential for successful management with limited intervention. This case contributes to the existing literature by illustrating a clinically relevant presentation and reinforcing the need for increased awareness of RDD in the differential diagnosis of pediatric lymphadenopathy.

## Case presentation

An 11-year-old boy was referred for evaluation of progressive left cervical lymphadenopathy, evolving over one month. There was no significant past medical or family history. The lymphadenopathy was painful and associated with fever and asthenia, without weight loss, night sweats, or hemorrhagic manifestations. These features initially raised concern for infectious or malignant etiologies, particularly tuberculosis and lymphoma, which are common causes of persistent lymphadenopathy in this age group.

**Figure 1:**
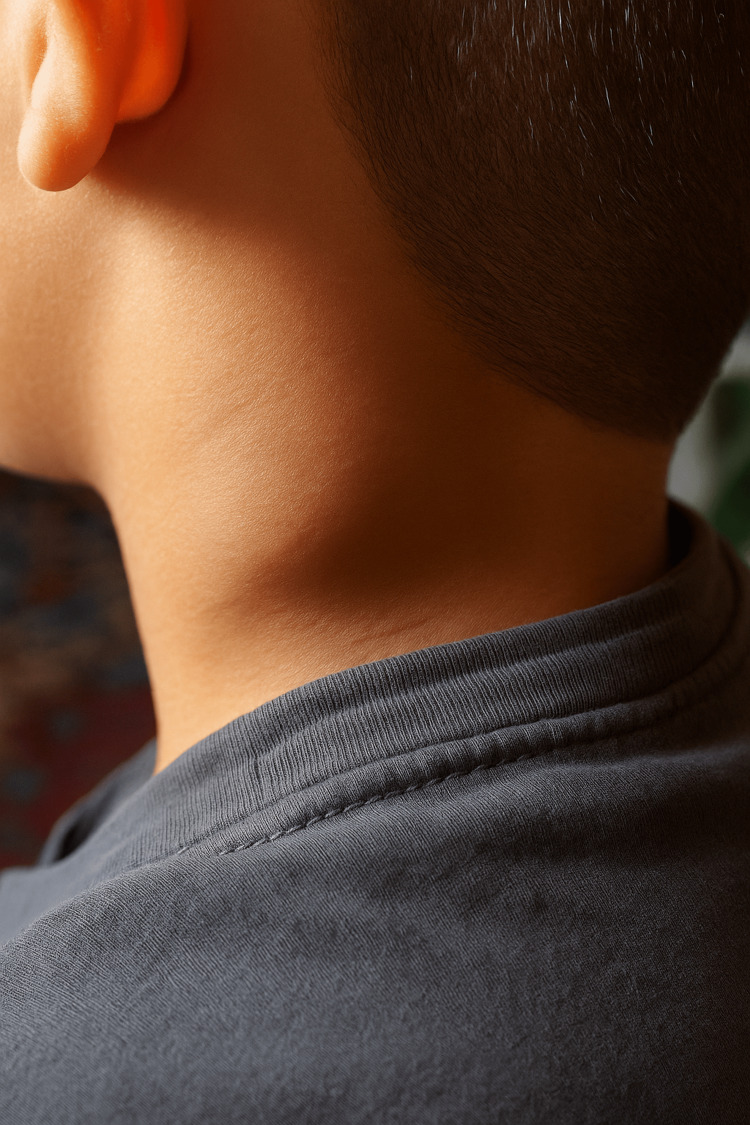
Photo of the patient showing cervical lymphadenopathy

On physical examination, the child was in good general condition, with stable hemodynamic and respiratory parameters. Cervical examination revealed a firm, non-inflammatory left cervical lymph node measuring approximately 3 cm in diameter (Figure 1). No additional lymphadenopathy, hepatomegaly, or splenomegaly was detected. The remainder of the clinical examination was unremarkable. A course of empiric antibiotics had been prescribed prior to referral, without clinical improvement, further supporting the need for etiological investigation.

**Figure 2:**
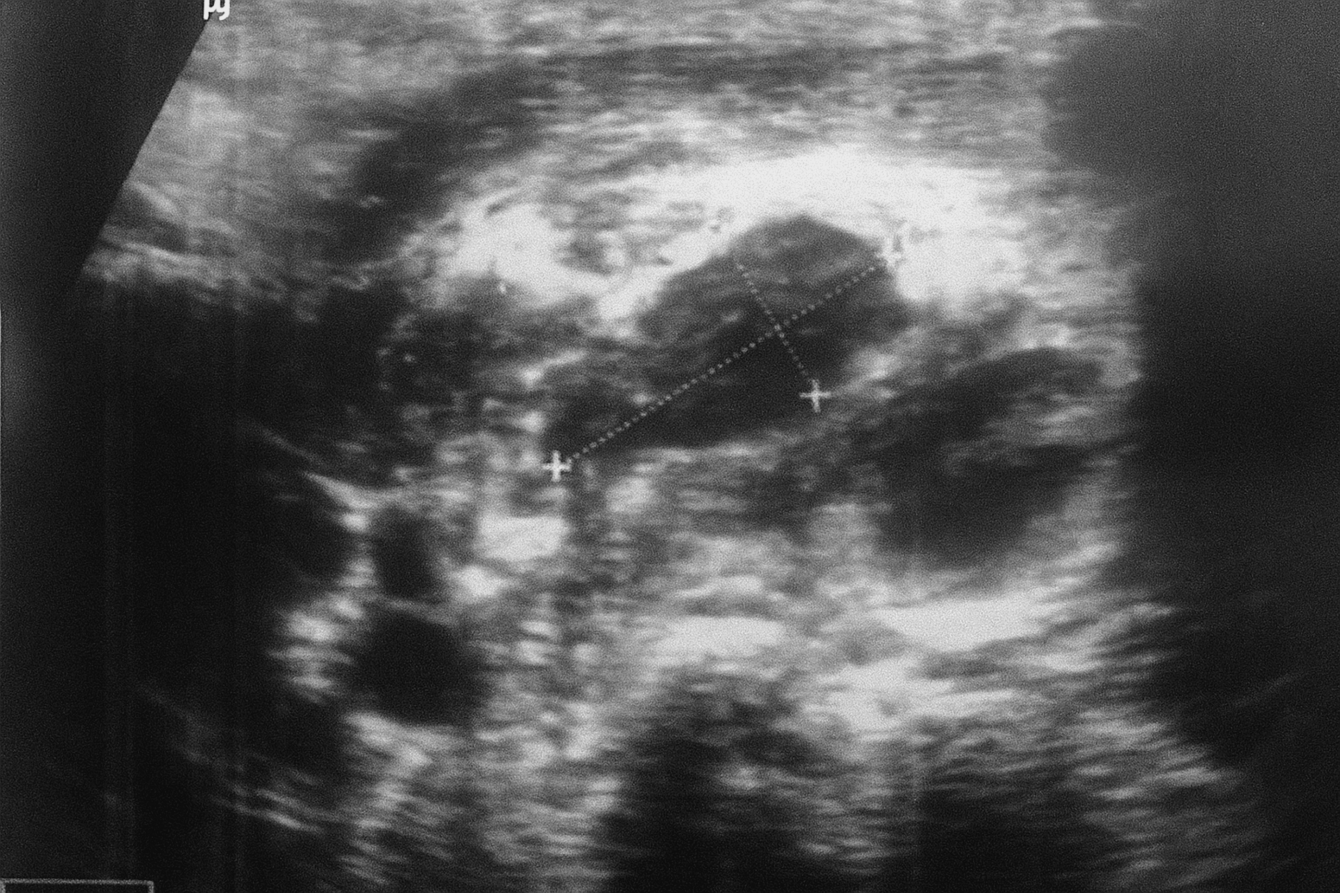
Cervical ultrasound showing multiple enlarged left cervical lymph nodes The largest node demonstrates partial liquefaction while retaining a predominant solid component, measuring 17.6 × 8 mm. This mixed solid-liquid appearance, in the absence of specific features of abscess or malignancy, supported the decision to proceed with excisional biopsy for definitive diagnosis.

**Figure 3:**
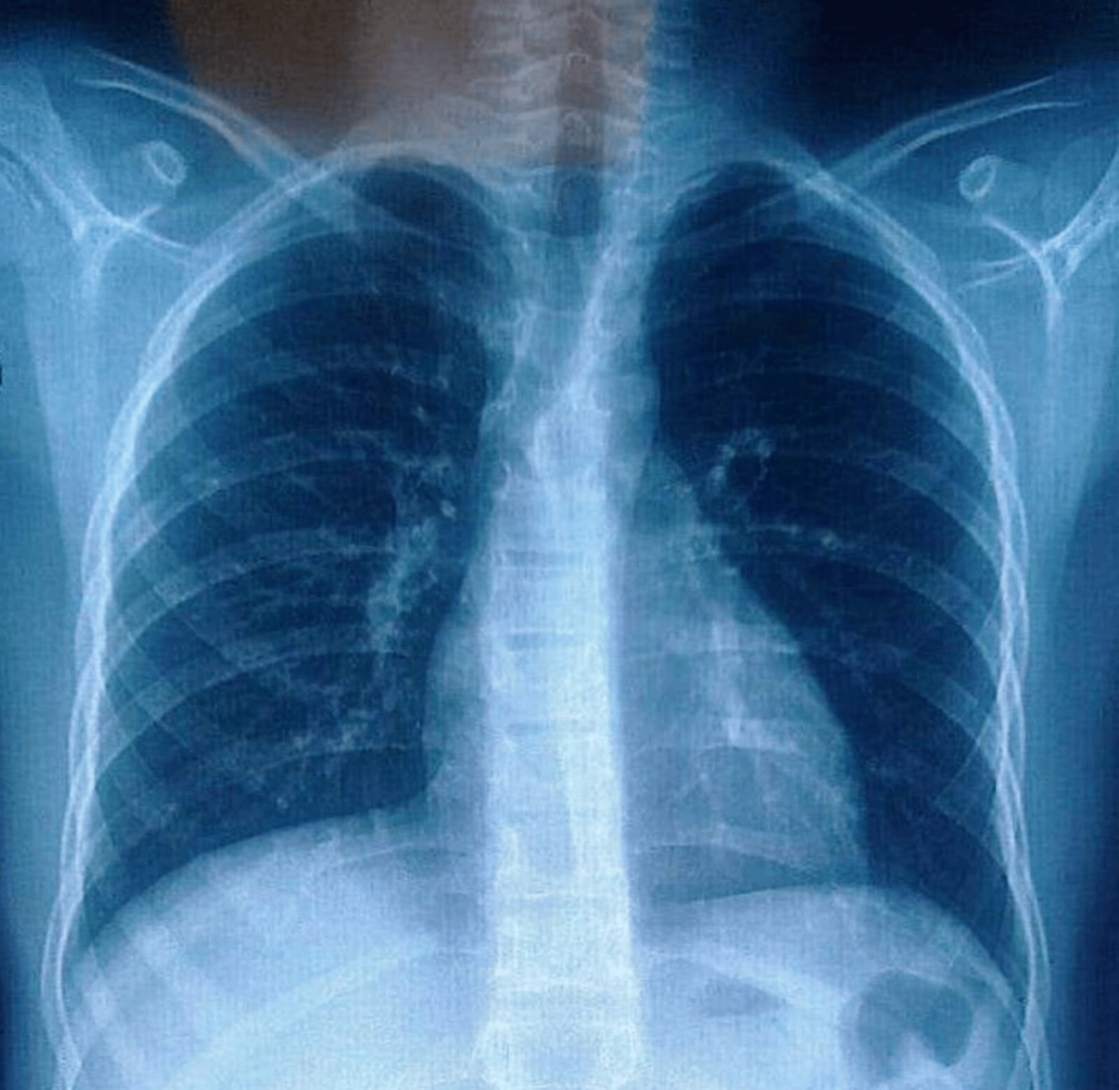
Chest X-ray in PA view showing no abnormalities

Cervical ultrasound was performed to assess the nature of the lymphadenopathy and demonstrated multiple enlarged left cervical lymph nodes, the largest showing partial liquefaction with a predominant solid component, measuring 17.6 × 8 mm (Figure 2). These findings did not allow differentiation between infectious, inflammatory, or neoplastic causes. A chest X-ray was obtained to screen for pulmonary tuberculosis and mediastinal involvement and was normal (Figure 3).

Laboratory investigations revealed mild neutrophilic leukocytosis, thrombocytosis, and elevated inflammatory markers (erythrocyte sedimentation rate and C-reactive protein), supporting an inflammatory process but lacking specificity. Lactate dehydrogenase levels were within normal limits, making high-grade lymphoma less likely but not excluding it. Pre-anesthetic laboratory tests were normal (Table 1). Given the persistence of lymphadenopathy and inconclusive noninvasive investigations, a lymph node biopsy was indicated for definitive diagnosis.

**Table 1:** Results of the laboratory investigations

Parameter	Result	Reference range
Complete blood count		
Red blood cell count	4.20 M/mm³	3.9-5.3 M/mm³
Hemoglobin	12 g/dL	12-16 g/dL
White blood cell count	12,090/mm³	4,000-10,000/mm³
Neutrophils	8,016/mm³	2,100-5,900/mm³
Lymphocytes	3,216/mm³	1,700-5,100/mm³
Monocytes	592/mm³	150-650/mm³
Eosinophils	254/mm³	50-350/mm³
Basophils	12/mm³	15-65/mm³
Platelet count	446,000/mm³	150,000-400,000/mm³
Other laboratory tests		
Erythrocyte sedimentation rate	50 mm/h	<7 mm/h
C-reactive protein	5 mg/dL	<10 mg/dL
Lactate dehydrogenase	193 U/L	≤278 U/L
Prothrombin time test	81.50%	70-100%
Urea	0.30 g/L	0.1-0.50 g/L
Creatinine	7.5 mg/L	6.6-10.9 mg/L
HIV serology 1 and 2	0.016	<1
Hepatitis B serology	0.664	<1
Hepatitis C serology	0.664	<1
Antinuclear antibodies	<80	<80
Rheumatoid factor	<10	<10

**Figure 4:**
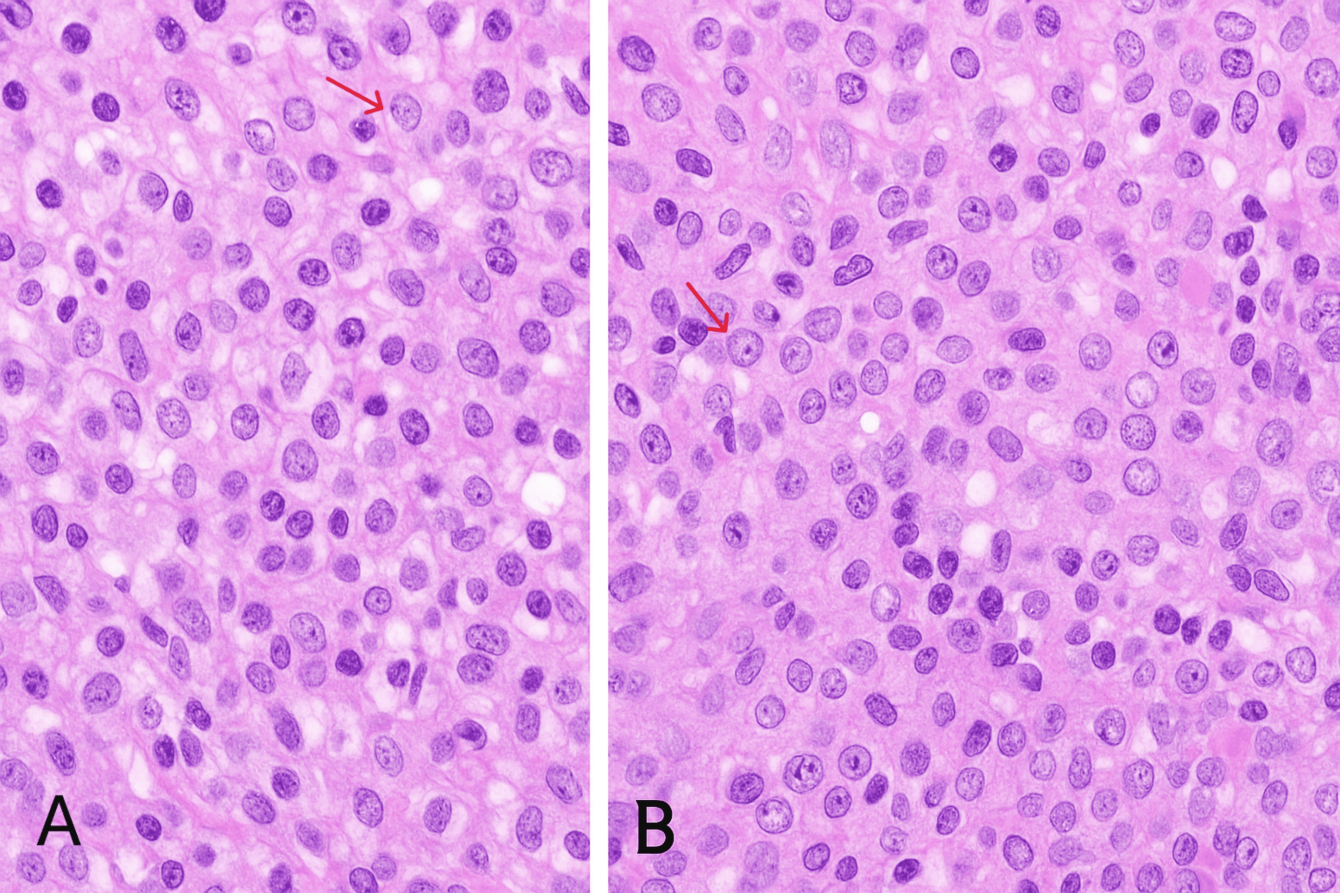
(A, B) Microscopic sections of nodal RDD showing proliferation of histiocytes: large, pale cells with abundant cytoplasm and central nucleoli RDD, Rosai-Dorfman disease

The patient underwent a cervicotomy with excisional biopsy. Histopathological examination revealed a polymorphic inflammatory infiltrate composed of lymphocytes, plasma cells, and neutrophils. Numerous large histiocytic cells with irregular nuclei, vesicular chromatin, prominent nucleoli, and abundant eosinophilic cytoplasm were observed, exhibiting emperipolesis, a characteristic feature of RDD. No granulomas or caseous necrosis were identified, arguing against tuberculosis (Figure 4).

**Figure 5:**
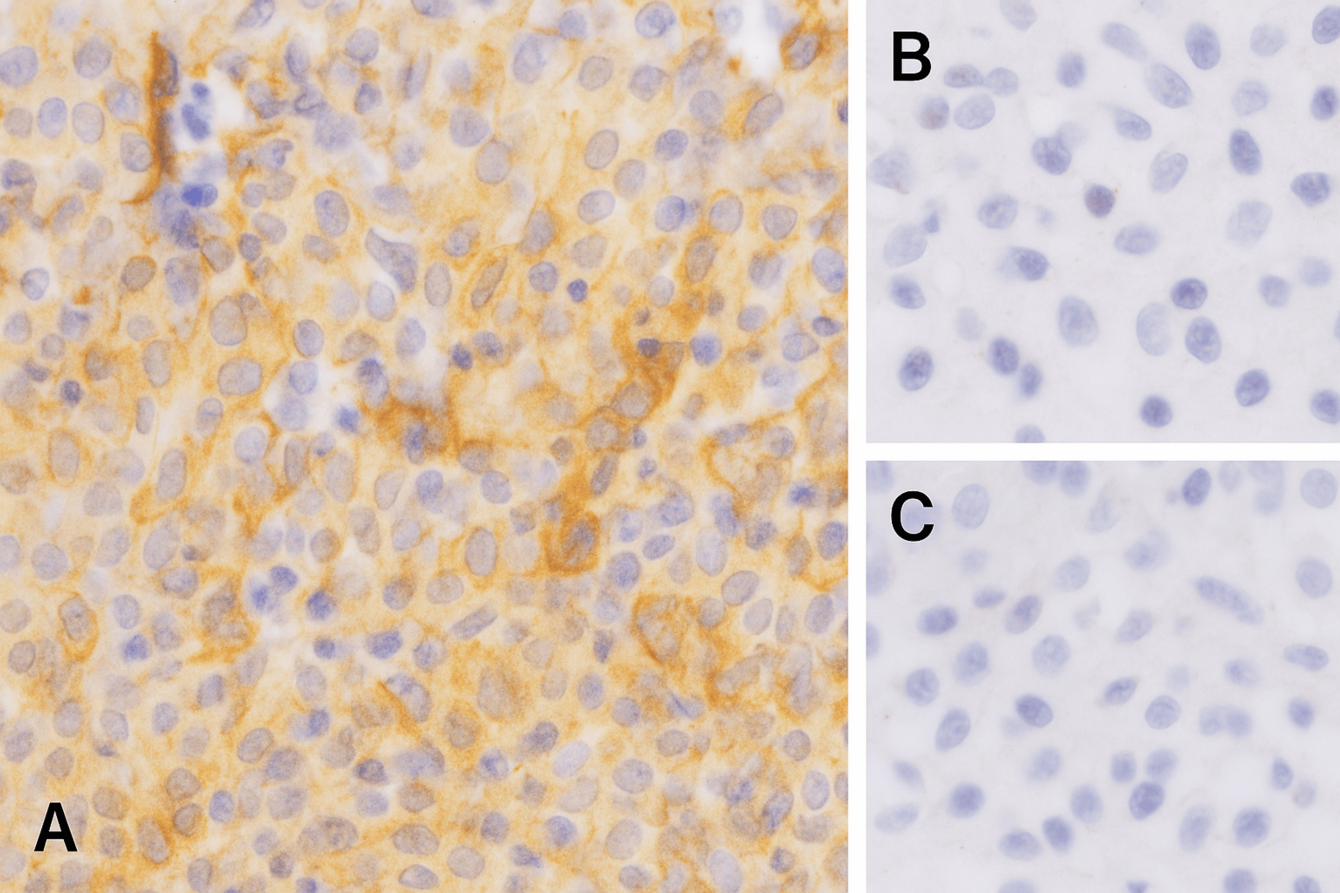
Immunohistochemical staining showing CD163 positivity in large histiocytic cells (A), with negative CD20 (B) and CD1a (C) expression This profile confirms the histiocytic nature of the lesion and supports the diagnosis of RDD while excluding lymphoma and LCH. LCH, Langerhans cell histiocytosis; RDD, Rosai-Dorfman disease

Immunohistochemical analysis demonstrated CD163 positivity, confirming the histiocytic nature of the large cells, and negativity for CD1a, excluding LCH. Additional markers, including CD20, CD5, CD15, CD30, and PAX5, were negative, ruling out lymphoid malignancies (Figure 5). Although S100 immunostaining, frequently positive in RDD, was not performed in this case, the diagnosis was supported by the typical morphological features and immunophenotypic profile in conjunction with the clinical presentation.

Further investigations, including a thoraco-abdominal computed tomography scan, were performed to assess for extranodal or systemic involvement and were unremarkable. Viral serologies (HIV, HBV, and HCV) and autoimmune markers were negative (Table 1), supporting an isolated form of the disease.

Based on these findings, a diagnosis of RDD with isolated cervical lymph node involvement was established. Given the localized disease and absence of systemic symptoms, no additional medical treatment was initiated. The diagnosis and favorable prognosis were explained to the patient’s family, and regular clinical follow-up was arranged. At six months, the patient remained asymptomatic, with no evidence of recurrence or complications.

The timeline of clinical events is presented in Table 2.

**Table 2:** Timeline of clinical events RDD, Rosai-Dorfman disease

Time point	Clinical event/findings
Month 1	Onset of left cervical lymphadenopathy associated with fever and asthenia
Prior to admission	Empiric antibiotic therapy with no clinical improvement
Day 25	Cervical ultrasound showing multiple enlarged left cervical lymph nodes with partial liquefaction
Admission	Clinical evaluation; laboratory investigations showing elevated inflammatory markers; normal chest X-ray
Day 0	Excisional lymph node biopsy via cervicotomy
Postoperative period	Histopathological and immunohistochemical findings consistent with RDD
Follow-up (six months)	No recurrence or complications

## Discussion

Epidemiology

RDD is a rare condition, with a prevalence of 1 in 200,000 and an estimated 100 new cases per year in the United States [4]. It predominantly affects individuals in their second or third decade of life, with a higher incidence in males and in individuals of African descent. Although RDD can occur in both children and adults, the median age at diagnosis is 20.6 years [4,6,7].

In this context, the occurrence of RDD in an 11-year-old boy further emphasizes the rarity of pediatric presentations and highlights the importance of maintaining clinical awareness of this condition in younger patients.

Etiopathology

The exact cause of RDD remains unclear, but possible contributing factors include infectious agents, immune system disorders, and genetic mutations such as *SLC29A3*. RDD and LCH share a common bone marrow precursor, and in some cases, patients treated for LCH have later developed RDD. Additionally, RDD has been associated with leukemia, Hodgkin lymphoma, and non-Hodgkin lymphoma [2,4,9,10].

Although no molecular testing was performed on our patient, the absence of systemic involvement and the favorable clinical course are consistent with reports suggesting biological heterogeneity within RDD. This heterogeneity may partly explain the wide spectrum of clinical presentations and outcomes reported in pediatric patients.

Symptoms

In pediatric patients, RDD often presents with a nonspecific constellation of signs and symptoms, which can complicate timely diagnosis. Disease manifestations may be unifocal or multisystemic, with combined nodal and extranodal involvement frequently reported [4,6,7,9-13]. Cervical lymphadenopathy in RDD typically presents as bilateral and generalized, while isolated nodal disease accounts for only a minority of cases (approximately 5%) [14].

In contrast to the typical bilateral presentation described in the literature, our patient exhibited isolated unilateral cervical lymphadenopathy without extranodal involvement. This underscores an atypical clinical pattern that may delay diagnosis or mimic more common conditions. Such an atypical presentation raises the question of whether isolated unilateral nodal disease in children represents a distinct and underrecognized clinical pattern of RDD, with potential implications for diagnostic strategies and management decisions.

Differential diagnoses

A thorough evaluation of differential diagnoses is essential, as RDD shares clinical and histological features with a broad spectrum of disorders [7]. Histiocytic diseases, lymphoproliferative malignancies, infectious conditions, and inflammatory disorders must all be considered [2,3,15,16].

In our case, the combination of persistent lymphadenopathy, fever, and inflammatory laboratory findings initially raised suspicion for tuberculosis and lymphoma, which are common considerations in pediatric practice. This diagnostic pathway illustrates the practical challenges clinicians face when encountering atypical presentations of RDD.

Immunohistochemistry is decisive: RDD typically demonstrates CD1a-, S100+, and emperipolesis [2]. In our patient, immunohistochemistry confirmed RDD with CD163+, CD20-, and CD1a- histiocytes (Figure 5), ruling out tuberculosis and lymphoma.

Laboratory tests

Laboratory abnormalities are common in RDD and include elevated ESR, leukocytosis, and thrombocytosis [4,6]. Our patient demonstrated a laboratory profile consistent with these nonspecific inflammatory changes, reinforcing the limited diagnostic specificity of laboratory testing and the importance of histopathological confirmation.

Imaging

Imaging plays a crucial role in evaluating disease extent and excluding extranodal involvement [4,8]. In our patient, the absence of systemic or extranodal disease on imaging supported a localized form of RDD, which has been associated with a more favorable prognosis.

Histopathology

Diagnosis is established by biopsy and immunohistochemistry, with emperipolesis representing a hallmark feature [2,3,11]. In this case, the presence of typical morphological features combined with a compatible immunophenotype allowed for a confident diagnosis despite the absence of S100 staining, which is acknowledged as a limitation.

Treatment

No standardized treatment exists, as RDD is often self-limiting [4,12]. Management strategies range from observation to systemic medical therapy or surgical intervention, depending on disease extent and symptom burden. In the present case, surgical excision alone was considered appropriate given the localized nodal involvement, absence of systemic disease, and favorable postoperative course, supporting a conservative therapeutic approach in selected pediatric patients.

Prognosis

Prognosis in pediatric RDD remains variable, although nodal disease generally follows a benign course [4,6,12]. At six months of follow-up, our patient showed no evidence of recurrence, consistent with the favorable outcomes reported in similar localized pediatric cases.

This case adds to the limited literature on pediatric RDD presenting as isolated unilateral cervical lymphadenopathy, a presentation reported in only a small subset of patients. It highlights the diagnostic challenges posed by nonspecific clinical, laboratory, and imaging findings and underscores the importance of histopathological examination in establishing the diagnosis. By illustrating a diagnostic pathway that initially favored more common pediatric conditions such as tuberculosis and lymphoma, this report reflects real-world clinical decision-making and reinforces the need to include RDD in the differential diagnosis of persistent cervical lymphadenopathy in children.

Despite being a well-described entity, RDD remains underrecognized in pediatric practice. This case is clinically relevant, as it demonstrates that limited surgical intervention may be sufficient in selected patients with localized disease, thereby preventing overtreatment. Increased awareness of atypical presentations, such as unilateral nodal involvement, may facilitate earlier diagnosis, reduce unnecessary investigations, and guide appropriate management strategies in pediatric patients.

## Conclusions

This case underscores an atypical presentation of RDD in a pediatric patient, manifesting as isolated unilateral cervical lymphadenopathy, a pattern that may closely mimic more common infectious or malignant conditions. Such presentations can delay diagnosis and expose children to unnecessary investigations or treatments if the disease is not considered early in the diagnostic process. Our findings highlight the pivotal role of histopathological examination and immunohistochemistry in establishing an accurate diagnosis. Importantly, this report demonstrates that surgical excision alone may be sufficient for disease control in selected pediatric patients with localized nodal involvement and no systemic disease, supporting a conservative and individualized management approach. Beyond its immediate clinical implications, this case contributes to the growing recognition of the heterogeneity of RDD in children. Increased awareness of unilateral and localized presentations is essential, and the future incorporation of molecular testing may further improve diagnostic precision and help refine therapeutic decision-making in pediatric RDD.
